# Cancer patients’ knowledge about their disease and treatment before, during and after treatment: a prospective, longitudinal study

**DOI:** 10.1186/s12885-018-4164-5

**Published:** 2018-04-03

**Authors:** Ola Berger, Bjørn Henning Grønberg, Jon Håvard Loge, Stein Kaasa, Kari Sand

**Affiliations:** 1 Department of Clinical and Molecular Medicine, Faculty of Medicine and Health Sciences, NTNU, Norwegian University of Science and Technology, N-7491 Trondheim, Norway; 2 Cancer Clinic, St. Olavs hospital, Trondheim University Hospital, Trondheim, Norway; 3 European Palliative Care Research Centre (PRC), Department of Oncology, Oslo University Hospital and Institute of Clinical Medicine, University of Oslo, Oslo, Norway; 4 Institute of Basic Medical Sciences, Faculty of Medicine, University of Oslo, Oslo, Norway

**Keywords:** Patient education, Knowledge, Cancer, Breast cancer, Prostate cancer, EORTC QLQ-INFO25

## Abstract

**Background:**

Knowledge about disease and treatment is necessary before patients can consent to treatment. One of the few established instruments for evaluating whether sufficient information has been provided, is the EORTC QLQ-INFO25 questionnaire which was developed to measure how patients perceive information. The aim of this study was to investigate whether cancer patients’ level of knowledge about their disease and treatment was associated with their perception of and satisfaction with the information.

**Methods:**

Breast cancer patients referred for adjuvant chemotherapy and prostate cancer patients referred for curative radiotherapy were included. Level of knowledge about their disease and treatment was measured using study-specific questionnaires. Patients’ perception of and satisfaction with the received information was assessed using EORTC QLQ-INFO25. Assessments were done before the first consultation with an oncologist (T1), after the consultation (T2) and 8 weeks after start of treatment (T3).

**Results:**

Ninety eight patients were enrolled. Patients with higher education, daily Internet access and in paid employment had the highest baseline knowledge scores. The mean knowledge score increased significantly (T1: 16.4; T2: 20.8; T3: 21.3; *p* < 0.001.). During the same period, the patients reported on the INFO25 a significant, positive increase in how much information they had received, and that they were more satisfied with the information.

**Conclusions:**

Patients’ knowledge increased significantly during the study period, and they reported that they felt better informed and were more satisfied with the information, suggesting that EORTC QLQ-INFO25 might be used to evaluate cancer patients’ level of knowledge about their disease and treatment.

**Trial registration:**

ClinicalTrials.gov identifier: NCT01699672. Date of registration: September 21, 2012.

## Background

Patients should receive information about their disease, potential benefits and side effects of the proposed therapy, and give their consent before treatment commences. Relevant and understandable information is a prerequisite for patients to acquire enough knowledge to enable them to be actively involved in shared decision making, to comply with the treatment plan, to make them aware of potential side-effects and to understand what to do if side-effects occur. Furthermore, well informed patients are more satisfied with care [1], have a better sense of control of their total situation [2], and report a better quality of life [3].

There are several potential barriers to provision of relevant and understandable information. Medical information has become more complex [4, 5], health care personnel’s (HCP) communication skills vary [6], written, standardised information is not always available, there is not always enough time, and interruptions are frequent [6, 7]. Moreover, fatigue, distress and anxiety are common among cancer patients [8]. Even when adequate information is provided, patients do not necessarily feel adequately informed or satisfied with the information [6, 7, 9], or gain enough knowledge to make treatment decisions or to follow instructions from health care personnel. This probably explains why approximately half of the complaints from patients and relatives to the Health and Social Services Ombudsman in Norway concerns poor communication between HCP, patients and relatives [10]. An important challenge for HCP when providing information is that there is no established method for evaluating whether patients are well informed or satisfied with the information they have been given.

The European Organization for Research and Treatment of Cancer (EORTC) QLQ-INFO25 is a relatively new 25 item self-report questionnaire developed to measure how cancer patients perceive the information they receive. It measures how much information patients perceive to have received about their disease, medical tests, treatment, help and support available; whether written or audio-visual information has been provided; whether they are satisfied with the amount of information; whether they would like to receive more or less information; and whether the information has been helpful. The EORTC QLQ-INFO25 has been used in studies of patients with various cancer types, e.g. breast, prostate, gynaecological and haematological, and patients from several different countries (Sweden, Spain, Germany, United Kingdom, Austria and Taiwan) and has proven to have good internal consistency and good test-retest reliability [11–14]. Given the construct of QLQ-INFO25, the scores may reflect the level of knowledge about disease and treatment, but no studies have investigated whether this is the case.

## Methods

### Aims of the study

The aims of this study were to compare cancer patients’ level of knowledge about their disease and treatment before and after consultation with an oncologist with their perception of and satisfaction with the information they had received.

### Design and setting

This is a prospective, longitudinal study. Breast cancer patients referred to the Cancer Clinic, St. Olavs Hospital, Trondheim University Hospital in Norway for adjuvant chemotherapy (six courses of 5-FU, epirubicin and cyclophosphamide followed by eight weeks of taxanes) after surgery, and patients with prostate cancer referred for curative radiotherapy (78 Gy in 39 fractions) were eligible if they were 18 years or older and gave written informed consent. Thus, all patients had been diagnosed with cancer and had received some information about their disease and treatment before they were referred to our clinic.

### Measures of knowledge about disease and treatment – Development of questionnaires

We were not able to identify a standard method for assessing patients’ level of knowledge since the relevant information depends on the type of cancer and treatment regimen. Thus, we developed two study-specific questionnaires for the level of knowledge – one for the breast cancer patients, and one for the prostate cancer patients.

These questionnaires were developed as follows: First, we collected information material from other Nordic hospitals (University Hospital of North Norway and Stavanger University Hospital in Norway, Karolinska University Hospital and Uppsala University Hospital in Sweden and Rigshospitalet in Denmark) and from brochures and webpages by the Norwegian Cancer Society, the Norwegian Prostate Cancer Society, Society for Breast Cancer, and from Oncolex, a web-site developed by Oslo University Hospital to provide information for cancer patients and relatives [15–17].

Secondly, from these sources, the first author (OB) identified 109 information elements about breast cancer and treatment and 80 information elements about prostate cancer and treatment as a basis for a consensus regarding items to include the questionnaires. We considered 25 items sufficient for assessing the patients’ knowledge, while at the same time ensuring a high completion rate [18, 19]. A two-round Delphi process was conducted to reduce the number of elements from 109/80 to 25 for each questionnaire.

In the first round, an expert group of researchers and physicians treating breast and prostate cancer patients at our clinic were asked to select the 25 items (out of the 109/80) they considered most relevant for assessment of patients’ knowledge about disease and treatment before commencing therapy, to add missing items, and to comment on the wording. After the first round, the number of items was reduced to 33 for the prostate cancer knowledge questionnaire (PKQ) and 30 for the breast cancer knowledge questionnaire (BKQ). In the second round, the expert group was again asked to select the 25 most relevant of the remaining items, and to provide comments as in the first round. Based on the feedback, the first version of the two 25-item questionnaires was constructed.

The two questionnaires were then reviewed by ten nurses and ten patients at our clinic with respect to readability and comprehensibility, and adjusted according to their comments. The final versions are shown in Table 1. Correct answers scored 1 point, incorrect answers or “Don’t know” scored 0. The scores were added to a maximum of 25.

**Table 1:** Questionnaires used for measuring knowledge about breast and prostate cancer - and cancer treatment. Correct answers are indicated with an “X”

A Breast cancer knowledge questionnaire (BKQ)				% correct answers		
We are interested in your knowledge about breast cancer. The questionnaire below contains statements about breast cancer and its treatment. We kindly ask you to answer whether you believe they are true, false or you do not know.						
	Statements about breast cancer:	TRUE	FALSE	T1	T2	T3
1	Breast cancer is the most common type of cancer among women in Norway?	X		79%	81%	86%
2	We know for sure that diet could influence the chances of developing breast cancer?		X	95%	100%	100%
3	New lumps in the breast among young women varying in size with the menstrual cycle, are most probably breast cancer?		X	44%	60%	66%
4	In most cases, breast cancer causes no symptoms and is detected by mammography screening?	X		19%	61%	74%
5	Tissue analyses can reveal whether a breast cancer tumor has hormone receptors?	X		98%	97%	100%
6	Breast cancer usually occurs in the breasts’ glandular tissue?	X		77%	89%	88%
7	Chemotherapy only affects cancer cells?		X	91%	100%	100%
8	It is completely safe to use alternative therapies during chemotherapy for breast cancer?		X	58%	86%	97%
9	To avoid infections during chemotherapy, I should stay at home as much as possible?		X	67%	69%	76%
10	The chemotherapy is given every 3 weeks?	X		100%	100%	100%
11	Chemotherapy prevents cell division?	X		37%	78%	79%
12	During chemotherapy I can eat what I want?	X		23%	78%	65%
13	Radiotherapy causes pain during the irradiation?		X	70%	86%	91%
14	Radiotherapy only affects cancer cells and not normal cells?		X	81%	89%	91%
15	Smoking can reduce the efficacy of radiotherapy?	X		56%	100%	100%
16	Radiotherapy is given each weekday except Saturdays, Sundays and public holidays for 5–6 weeks?	X		79%	94%	94%
17	Hormone therapy increases the amount of female hormones in the body and thus inhibits growth of breast cancer cells?		X	88%	94%	100%
18	Hormonal therapy is recommended for 5 years after you have removed your breast?	X		40%	44%	53%
19	Fever during chemotherapy is normal and not something you should be concerned about		X	49%	97%	100%
20	Is it recommended to avoid antiemetics until you become nauseous?		X	49%	97%	94%
21	Nausea usually occurs immediately after the chemotherapy is administered and lasts for 2 days?		X	67%	81%	94%
22	During chemotherapy I should avoid drinking much since this can reduce the effect of the treatment?		X	72%	97%	97%
23	Fever during chemotherapy may be a symptom of severe side-effects and I should immediately contact emergency services or the cancer clinic?	X		67%	86%	91%
24	Fever is most accurately measured in the rectum?	X		60%	72%	79%
25	Side effects of radiotherapy occur immediately after the first treatment and usually resolve the day after the last treatment?		X	30%	69%	100%
	Overall			62%	84%	85%
B Prostate cancer knowledge questionnaire (PKQ)				% correct answers		
We are interested in your knowledge about prostate cancer. The questionnaire below contains statements about prostate cancer and its treatment. We kindly ask you to answer whether you believe they are true, false or you do not know.						
	Statements about prostate cancer:	TRUE	FALSE	T1	T2	T3
1	Prostate cancer is the most common type of cancer in Norway?		X	96%	98%	94%
2	Prostate cancer is the most common type of cancer among men in Norway?	X		91%	92%	92%
3	We know for sure that your diet can affect the chances of developing prostate cancer?		X	91%	92%	90%
4	A thin and weak urinary stream could be a symptom of prostate cancer?	X		96%	98%	100%
5	Anemia could be a symptom of prostate cancer?	X		91%	98%	96%
6	Nocturnal urination could be a symptom of prostate cancer?	X		88%	94%	94%
7	An elevated PSA-level means having prostate cancer?		X	91%	92%	91%
8	The radiotherapy starts the same day as the CT planning scan is performed?		X	25%	65%	52%
9	The urethra passes through the prostate gland?	X		39%	61%	60%
10	The prostate gland is located directly in front of the rectum?	X		68%	92%	100%
11	It is not a problem to skip radiotherapy for a week?		X	58%	73%	87%
12	Radiation therapy only affects cancer cells and not normal cells?		X	96%	100%	100%
13	Gold grains are inserted into the prostate gland because it can have a healing effect on the cancer?		X	96%	98%	96%
14	During the radiotherapy I should avoid drinking too much because it can cause unnecessary bladder irritation?		X	56%	71%	62%
15	During radiotherapy I should stay as sedentary as possible and avoid physical activity?		X	86%	98%	98%
16	Alcohol should be avoided during the treatment period?		X	84%	86%	94%
17	Smoking can reduce the efficacy of radiotherapy?	X		27%	63%	81%
18	It is completely safe to use alternative therapies during radiotherapy for prostate cancer?		X	1%	12%	25%
19	The purpose of adding hormonal therapy to radiotherapy is to slow the growth of prostate cancer cells?	X		36%	76%	62%
20	Side effects of radiotherapy occur immediately after the first treatment day and usually resolve the day after the last treatment day?		X	73%	90%	87%
21	Diarrrhea and mucous stools is a common side effect of radiotherapy?	X		48%	80%	83%
22	There is no treatment for radiotherapy-induced diarrhea?		X	71%	78%	92%
23	During radiotherapy, the skin may become sore in the area being treated?		X	38%	94%	100%
24	Radiotherapy-induced pain and haematuria indicate that the treatment is ineffective?		X	55%	90%	75%
25	It is normal to be sore in the irradiated parts of the body a few weeks after the treatment has been completed?	X		39%	90%	91%
	Overall			66%	83%	84%

### Patients’ perception of information

EORTC QLQ-INFO25 questionnaire was developed to measure how cancer patients perceive the information they receive during treatment and follow-up and is, to our knowledge, the only validated questionnaire for this purpose [11, 20, 21]. QLQ-INFO25 consists of 25 items: information about disease (4 items), medical tests (3 items), treatment (6 items), other services (4 items) plus eight single items. For four items, patients answer yes or no. For the remaining items, the patients rate 1 = not at all, 2 = a little, 3 = quite a bit, or 4 = very much [11]. Scores are then transformed to a linear scale from 0 to 100 according to the EORTC scoring manual [22]. A higher score indicates better-perceived information.

### Study procedures

Study nurses screened all referral letters to the Cancer clinic and attended weekly multidisciplinary meetings to identify potential participants. They phoned eligible patients, informed about the study and asked if the patients were willing to receive a written request by mail. Informed consent document and the first questionnaires were mailed to those who agreed to join the study.

**Fig. 1:**
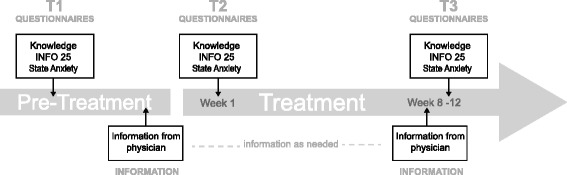
Shows the timing of questionnaires, appointments with information from physician and time frame of the treatment

The patients were asked to complete the PKQ or BKQ questionnaire and QLQ-INFO25 three times. The following socio-demographic data were registered at baseline: Age, education, use of Internet, work status, and relationship status. The baseline assessment was performed at home within one week before the first consultation with a physician at our clinic (T1). The second assessment was conducted at our clinic or at home within one week after this consultation (T2). The third assessment was conducted at the clinic or at home within one week after the second routine consultation with a physician (T3), approximately eight weeks after start of treatment. For breast cancer patients, T3 was before the fourth chemotherapy-course, for prostate cancer patients at the end of radiotherapy (Fig. 1).

The first consultation with a physician took place within one week before treatment started and lasted 45–60 min. Most breast cancer patients met with a resident, while most prostate cancer patients met with a Consultant in Oncology. Patients were informed about their disease, how chemo- and radiotherapy is administered and potential toxicity of the treatment, and when to contact health care personnel if side effects occurred. All patients were offered written information. Physicians were blinded to their patients’ participation in the trial.

Further information was provided by HCP who treated or followed the patients during treatment. A physician examined patients if side-effects occurred or upon the patients’ request.

### Statistical considerations

The sample size was calculated based on the experience from the pilot testing of the knowledge questionnaires. G-power for Mac version 3.1.9.2 was used. A baseline knowledge score of 18 points was expected, and we considered an improvement of two points to be clinically relevant. With an estimated standard deviation of 3.5 points, power of 90%, and alfa of 5%, 66 patients were required. To compensate for a drop-out rate of maximum 45%, we aimed at enrolling 96 patients.

The knowledge scores and QLQ-INFO25 scores were compared using the paired t-test [23]. The Mann-Whitney test was used for group comparisons. Significance level was defined as *p* < 0.05.

## Results

### Study participants

**Fig. 2:**
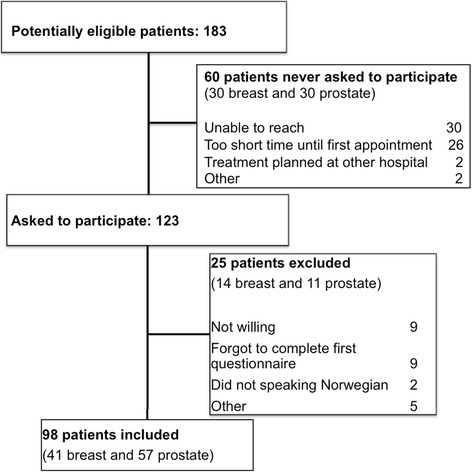
Patient selection

From November 2012 until November 2014, we identified 183 eligible patients. Of these, 85 were not enrolled due to the following reasons: We were not able to reach 30 patients before their first visit at our clinic, 26 patients had too short time until first appointment, two were not supposed to be treated at our clinic and two were not contacted for other reasons. Of the 123 patients asked to participate, nine declined, nine forgot to complete the first questionnaires, two did not speak Norwegian, and five were found ineligible after being contacted. Thus, 98 patients were included in the present study (Fig. 2).

Median age was 66 years, 58% had prostate cancer, 82% were in a relationship, 44% had a degree from college or university, 39% were paid employees, and 68% used Internet on a daily basis (Table 2). All patients received treatment as planned. Completion rate of the questionnaires varied between 90 and 100% (Tables 3 and 4).

**Table 2:** Patients characteristics

	All patients (*n* = 98)		Breast cancer patients (*n* = 41)		Prostate cancer patients (*n* = 57)	
	n	%	n	%	n	%
Age						
Median age (range)	66 (27–82)		57 (27–75)		73 (62–82)	
≥ 60 years	73	76%	18	44%	55	97%
Highest education						
Primary school 7–10 years	18	19%	9	22%	9	16%
High School	36	37%	12	29%	24	44%
Academy/university ≤3 years	19	20%	11	27%	8	15%
Academy/university > 3 years	23	24%	9	22%	14	25%
Unknown	2	2%	0	0%	2	3%
Use of internet						
Daily	65	68%	31	76%	34	60%
Weekly	16	17%	7	17%	9	16%
Monthly	1	1%	0	0%	1	2%
Never	13	14%	3	7%	10	19%
Unknown	3	3%	0	0%	3	5%
Work status						
Paid employment	38	39%	26	63%	12	21%
Pensioner in paid employment	2	2%	1	2%	1	2%
Pensioner	40	41%	5	12%	35	61%
Unemployed	1	1%	1	2%	0	0%
Disability benefit pensioner	2	2%	0	0%	2	4%
Disability benefit	9	9%	4	10%	5	9%
Unknown	6	6%	4	11%	2	3%
Relationship Status						
Married	66	69%	23	56%	43	78%
Cohabitant	13	13%	11	27%	2	4%
Single	17	18%	7	17%	10	18%
Unknown	2	2%	0	0%	2	3%

**Table 3:** Mean knowledge scores at T1, T2 and T3 for the whole study population and for subgroups including those with anxiety below and above the median anxiety level

	Knowledge Score T1 *n* = 98	p	Knowledge score T2 *n* = 88	p	Knowledge score T3 *n* = 88	p	Change in Knowledge score from T1 to T3 *n* = 88	p
All patients	16.4 (SD 4.6)		20.8 (SD 3.1)		21.3 (SD 2.6)		5.1 (SD 3.8)	
Cancer								
Breast	16.4 (SD 5.4)		21.1 (SD 3.4)		22.1 (SD 2.6)		6.4 (SD 4.1)	
Prostate	16.4 (SD 4.1)	0.68	20.7 (SD 2.9)	0.32	20.8 (SD 2.5)	0.010	4.3 (SD 3.4)	0.039
Relationship Status								
Married / Cohabitant	16.6 (SD 4.5)		20.8 (SD 3.2)		21.4 (SD 2.6)		5.0 (SD 3.9)	
Single	15.7 (SD 5.2)	0.53	20.9 (SD 2.9)	0.88	21.1 (SD 2.3) (*p* = 0.39).	0.39	5.9 (SD 3.6)	0.32
Internet use								
Daily	17.5 (SD 4.0)		21.2 (SD 3.0)		21.8 (SD 2.4)		4.6 (SD 3.4)	
Other	14.4 (SD 5.0)	0.005	20.4 (SD 3.1)	0.22	20.6 (SD 2.5)	0.022	6.3 (SD 4.4)	0.10
Education level:								
Lower (Primary school/ High School)	14.9 (SD 4.6)		20.1 (SD 2.5)		20.9 (SD 2.5)		6.0 (SD 3.8)	
Higher (Academy/University)	18.4 (SD 3.9)	< 0.001	22.0 (SD 2.5)	0.034	22.0 (SD 2.5)	0.022	4.1 (SD 3.7)	0.043
Work status								
Paid employment	17.3 (SD 4.7)		21.6 (SD 2.8)		22.3 (SD 2.3)		5.3 (SD 4.4)	
Other	15.8 (SD 4.6)	0.09	20.2 (SD 3.4)	0.030	20.5 (SD 3.4)	< 0.001	5.0 (SD 3.3)	0.98
State Anxiety Level at baseline								
Below median anxiety score (34.0)	16.4 (SD 4.3)		20.6 (SD3.3)		21.3 (SD 2.6)		4.9 (SD 3.6)	
Above median anxiety score (34.0)	16.2 (SD 5.1)	0.80	21.3 (SD 2.8)	0.36	21.2 (SD 2.7)	0.97	5.6 (SD 4.4)	0.67

**Table 4:** Table shows different mean scores of QLQ-INFO25 subscales at T1, T2 and T3 for the total group, and for breast and prostate cancer patients respectively. All scores are shown with standard deviation (SD) and paired samples T-test *p*-values for mean at T1 compared to T3

	All patients				Breast				Prostate			
	T1 *n* = 98	T2 *n* = 88	T3 *n* = 88	*P*-value	T1 *n* = 41	T2 *n* = 38	T3 *n* = 36	*P*-value	T1 *n* = 57	T2 *n* = 49	T3 *n* = 52	*P*-value
Information about the disease	45.6 (SD 18.2)	56.2 (SD 19.1)	52.8 (SD 20.5)	0.004	50.6 (SD 16.5)	60.8 (SD 17.4)	56.4 (SD 21.6)	0.06	42.6 (SD 18.8)	52.6 (SD 19.7)	50.4 (SD 19.5)	0.024
Information about medical tests	59.7 (SD 22.0)	56.2 (SD19.1)	52.8 (SD 20.5)	0.015	62.9 (SD 22.4)	60.9 (SD 17.4)	56.4 (SD 21.6)	0.12	57.4 (SD 21.6)	52.6 (SD 19.7)	50.4 (SD 19.5)	0.06
Information about treatments	44.1 (SD 20.7)	68.4 (SD 16.5)	63.6 (SD 18.1)	< 0.001	39.2 (SD 20.0)	64.0 (SD 14.3)	64.4 (SD 16.7)	< 0.001	47.8 (SD 20.6)	71.8 (SD 17.5)	63.0 (SD 19.1)	< 0.001
Information about other services	14.2 (SD 17.5)	20.4 (SD 19.9)	19.6 (SD 19.5)	0.003	25.0 (SD 20.2)	26.8 (SD 19.4)	33.3 (SD 17.9)	0.032	6.2 (SD 9.2)	15.5 (SD 19.1)	10.1 (SD 14.2)	0.043
Information about different places of care	18.3 (SD 24.7)	20.7 (SD 27.0)	21.8 (SD 27.3)	0.24	21.1 (SD 25.6)	28.1 (SD 29.5)	31.5 (SD 27.5)	0.050	16.1 (SD 24.0)	15.0 (SD 23.6)	15.0 (SD 25.2)	0.89
Information about things you can do to help yourself	24.3 (SD 27.1)	34.9 (SD 32.5)	39.8 (SD 33.5)	< 0.001	37.4 (SD 27.1)	50.0 (SD 30.8)	53.7 (SD 35.0)	0.002	14.6 (SD 22.9)	23.1 (SD 29.0)	30.1 (SD 29.0)	0.001
Satisfaction with the information received	57.6 (SD 20.9)	78.4 (SD 20.9)	73.5 (SD 25.8)	< 0.001	59.4 (SD 26.4)	78.1 (SD 17.8)	76.9 (SD 22.3)	< 0.001	56.4 (SD 27.1)	78.7 (SD 23.1)	71.1 (SD 28.0)	0.001
Overall the information has been helpful	67.4 (SD 26.5)	85.2 (SD 17.4)	85.1 (SD 18.9)	< 0.001	73.3 (SD 22.9)	86.8 (SD 18.2)	87.3 (SD 16.4)	< 0.001	63.1 (SD 28.2)	84.0 (SD 16.8)	83.7 (SD 20.4)	< 0.001
Written information	72.6 (SD 44.8)	89.8 (SD 30.5)	84.1 (SD 36.8)	0.07	62.5 (SD 49.0)	97.4 (SD 16.2)	94.4 (SD 23.2)	0.003	30.1 (SD 29.0)	80.0 (SD 40.4)	84.0 (SD 37.0)	0.79
Wish to receive more information	68.8 (SD 46.6)	43.0 (SD 49.8)	34.9 (SD 47.9)	< 0.001	75.0 (SD 43.9)	48.7 (SD 50.7)	37.1 (SD 49.0)	< 0.001	64.3 (SD 48.3)	38.8 (SD 49.2)	33.3 (SD 47.6)	< 0.001
Wish you have received less information	0.0 (SD 0.0)	0.0 (SD 0.0)	0.0 (SD 0.0)	N/A	0.0 (SD 0.0)	0.0 (SD 0.0)	0.0 (SD 0.0)	N/A	0.0 (SD 0.0)	0.0 (SD 0.0)	0.0 (SD 0.0)	N/A
Global score	39.4 (SD 10.7)	46.4 (SD10.9)	44.2 (SD 11.2)	< 0.001	42.1 (SD 10.8)	50.4 (SD 9.5)	49.4 (SD 10.5)	< 0.001	37.6 (SD 10.2)	43.4 (SD 11.0)	40.6 (SD 10.3)	0.06
^*^Information on CD tape/ video	–	–	–	–	–	–	–	–	–	–	–	–

^*^Information on CD or video was not given to the patients and therefore not analyzed

### Patients’ level of knowledge

**Fig. 3:**
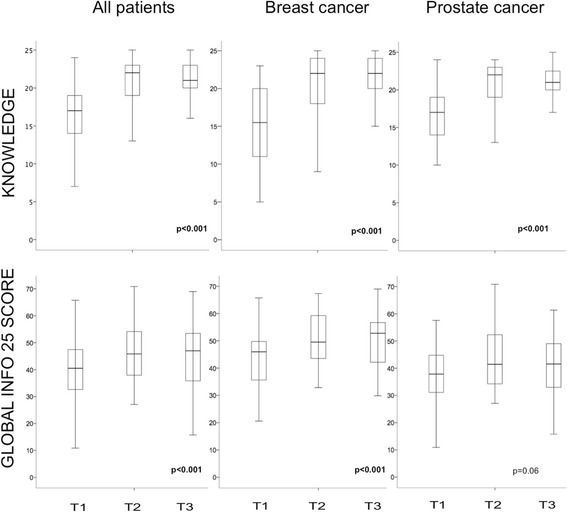
shows boxplot of knowledge (0–25) and global INFO25 (0–100) scores for all patients in total, breast and prostate cancer at T1, T2 and T3. *P*- values are student t- test between T1 and T3. Significant *P*- values in bold

Overall, the mean knowledge score was 16.4 points (range 5–23) at baseline (T1) (Fig. 3). There was no significant difference between breast and prostate cancer patients (breast cancer: 16.4, prostate cancer: 16.4; *p* = 0.68). Daily Internet use (yes: 17.5, no: 14.4; *p* = 0.005) and higher education (yes: 18.4, no: 14.9; *p* < 0.001) were associated with higher scores. There were no significant differences depending on marital status (relationship: 16.6, singles 15.7; *p* = 0.532) or paid employment (yes: 17.3, no: 15.8; *p* = 0.089) (Table 3).

**Fig. 4:**
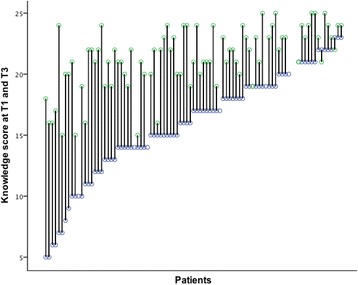
Dropline graph of all patients with lower circle showing knowledge score at T1 and upper circle knowledge at T3. Patients are sorted ascending from lowest to highest T1 knowledge score

Overall, the level of knowledge increased significantly from 16.4 points at T1 to 20.8 points at T2 and 21.3 points at T3 (*p* < 0.001) (Fig. 3). Figure 4 shows the change in score for each patient. All participants increased their score. Patients with the lowest baseline score increased their level of knowledge the most (Fig. 4).

At T3, breast cancer patients had a higher level of knowledge than prostate cancer patients (breast cancer: 22.1, prostate cancer: 20.8; *p* = 0.010). Other factors associated with higher scores at T3 were daily Internet use (yes: 21.8, no: 20.6; *p* = 0.022), higher education (yes: 22.0, no: 20.9; *p* = 0.022) and paid employment (yes: 22.1, no: 20.5; *p* < 0.001) (Table 3).

### Patients’ subjective perception of information measured on the QLQ-INFO25

From T1 until T3, there was a significant increase in the following QLQ-INFO25-scales: information about the disease (T1: 45.6, T3: 52.8; *p* = 0.004), information about treatment (T1: 44.1, T3: 63.6; *p* < 0.001), information about other services (T1: 14.2, T3: 19.6; *p* = 0.003), information about what patients can do to help themselves (T1: 24.3, T3: 39.8; *p* < 0.001), satisfaction with the received information (T1: 57.6, T3: 73.5; *p* < 0.001); overall helpfulness of the information (T1: 67.4, T3: 85.1; *p* < 0.001), and the global QLQ-INFO25 score (sum of all scales) (T1: 39.4, T3: 44.2; *p* < 0.001) (Table 4). During the study period, the proportion of patients who wished they had received more information, decreased from 68.8% at T1 to 34.9% at T3 (*p* < 0.001), and none wished they had received less information at any time point.

The proportion who reported to have received written information increased from 72.6% at T1 to 85% at T3 (Table 4). Significantly more prostate cancer patients reported to have received written information at T1 (prostate cancer: 80%, breast cancer: 62.5%; *p <* 0.01). Otherwise, there were no significant differences in QLQ-INFO25 scores between breast and prostate cancer patients (Table 4).

There was a significant decrease in patients’ perception of information about medical tests (T1: 59.7, T3 52.8; *p* = 0.015), and no significant change in information about different places of care (T1: 18.3, T3: 21.8; *p* = 0.24) (Table 4).

## Discussion

In this study of patients with breast and prostate cancer, we found a large inter-individual variation in the patients’ knowledge about their disease and the planned treatment before their first visit with an oncologist. Patients in paid employment, those accessing the Internet on a daily basis and those with a higher education had the highest baseline scores.

After receiving information from a physician, the patients’ average level of knowledge increased significantly to 21.3 out of 25 points (83%), possibly indicating that the knowledge acquired was sufficient for the patient to decide whether to consent to treatment. After 8–12 weeks of treatment, their level of knowledge increased slightly more to 21.4 out of 25 points. All patients, including those with the lowest baseline scores, improved their level of knowledge during the study period.

The increase in level of knowledge we observed in our cohort, is similar to what has been reported in other studies of cancer patients, e.g. breast cancer patients receiving information on an electronic tablet before adjuvant chemotherapy [5], prostate cancer patients offered a multimedia presentation before prostatectomy [24], and gastric cancer patients offered an interactive lecture after surgery [25]. A similar improvement in level of knowledge was also observed in studies of knowledge before and after intervention among patients with heart disease [26] and gastro oesophageal reflux disease [18]. These studies are, however, not necessarily comparable since the level of knowledge was measured using different questionnaires developed for each study, and different methods for informing patients were used [5, 19].

Parallel with the increasing level of knowledge, the patients reported a positive change in perception and satisfaction with the information they had received on 8 out of 10 scales of the QLQ-INFO25 (Fig. 4).

The QLQ-INFO25 scores increased significantly from T1 until T3, though the scores were slightly lower at T3 than at T2. Both the scale scores and the global INFO25 score at T3 was at the same level as the participants’ scores in the INFO25 validation study [11], in studies of breast cancer patients after receiving radiotherapy [12], prostate cancer patients after receiving radiotherapy or surgery [13] and Belgian cancer patients after receiving chemotherapy [14].

Considering that a change of INFO-25 scores of more than 10 point (10% on a 0–100 point scale) is considered clinically relevant [27, 28], the increase in score for “satisfaction on the information received” from 57,6 at T1 to 73,5 at T3 is large, statistically significant and clinically relevant. It is, however, unclear whether these scores reflect that patients have received sufficient information to make treatment decisions, since this is not measured on the INFO25, and there are no established cut-off values for INFO25 scores that can be used to assess whether patients have received sufficient information [11, 12].

The patients’ level of knowledge indicates that the patients in our study received and memorized the information considered most important by health care personnel, but it may still not be sufficient to cover the patients’ needs [29, 30]. Seventy three point five percent reported that they were satisfied with the information, and 34.9% patients reported that they wished more information. However, the study was not designed to assess which specific pieces of information the patients missed.

Main limitations of this study are the relatively small sample size and the high exclusion rate that could lead to a selection bias. No information was collected about the patients who did not consent to participation, and it is possible that we have included the best-motivated and well-informed patients. Still, the number of patients enrolled is higher than in other studies of knowledge in cancer patients [19], there was a substantial variation in baseline level of knowledge, and this is the first study to measure level of knowledge and perception of and satisfaction with information before, during and after cancer treatment.

Patients met different physicians, and in many cases, several physicians during the study period. The physicians did not receive any training before we started the study, and we did not assess what information that was actually given by the HCP or the interaction between HCP, patients and relatives, since the study was designed to evaluate current practice. Consequently, we do not know whether completing the knowledge questionnaires before the first consultation generated more questions from the patients and relatives.

We only enrolled patients receiving curative cancer therapy, since there is less heterogeneity with respect to treatment plans for patients with localized disease. It is possible that patients with more advanced disease would have been less able to comprehend and remember the information, responded differently on the INFO25, and would have been more interested in topics that were not included on the knowledge questionnaires. We did, for example, not measure the patients’ knowledge about the goals or efficacy of the planned treatment, alternative treatment options, long-term toxicities or prognosis. Some of these topics may be more important for patients receiving palliative therapy than patients with a high chance of cure. The knowledge questionnaires mainly reflected what HCP considered to be important knowledge about disease and planned treatment. We did not involve patient representatives when developing the questionnaires.

Furthermore, we do not know whether patients acquired information from other sources during the study period. Breast and prostate cancer patients are not necessarily comparable due to differences in gender, age and life situations. Women might be more prone to ask for information about their disease and treatment than men [9]. It is also possible that there was a learning effect of repeated completion of the questionnaires [19], causing our participants to reach a higher level of knowledge than non-participants.

The increase in level of knowledge was significant, and most patients reached a high level of knowledge. There is no objective measure of how much knowledge is required to consent to treatment, but a knowledge score of 20.8 out of 25 points is probably sufficient. However, 1/3 reported that they would like more information, and future studies should investigate what information patients miss, and whether missing information might influence treatment decisions.

Relevant information about cancer and treatment changes over time. Consequently, questionnaires or measuring knowledge such as our BKQ and PKQ are only relevant for a short time period. The positive change in QLQ-INFO25 scores that occurred in parallel with the increase in level of knowledge might suggest that QLQ-INFO25 can be used as a generic measure of knowledge across different cancer types, over time. This needs, of course, to be confirmed in future studies.

## Conclusion

We found that all patients acquired a higher level of knowledge about their disease and planned treatment after being informed by physicians and other health care personnel throughout their treatment period. This also applied to the patients with the lowest level of knowledge at baseline. The patients reported a significant improvement in perception and satisfaction with the information received during the study period.
